# Erratum to: Juvenile osteochondritis of femoral condyles: treatment with transchondral drilling. Analysis of 40 cases

**DOI:** 10.1007/s11832-014-0614-z

**Published:** 2014-10-08

**Authors:** Hayan Rammal, Philippe Gicquel, Ludovic Schneider, Claude Karger, Jean-Michel Clavert

**Affiliations:** Pediatric Orthopedic Surgery Department, Hautepierre Hospital, Moliere Avenue, 67098 Strasbourg Cedex, France

## Erratum to: J Child Orthop (2010) 4:39–44 DOI 10.1007/s11832-009-0225-2

In the original publication, the first and last names of all the authors have been interchanged and published incorrectly.

The correct names are as follows:

Hayan Rammal, Philippe Gicquel, Ludovic Schneider, Claude Karger, Jean-Michel Clavert.

